# Interactions between *Candida albicans* and *Enterococcus faecalis* in an Organotypic Oral Epithelial Model

**DOI:** 10.3390/microorganisms8111771

**Published:** 2020-11-11

**Authors:** Akshaya Lakshmi Krishnamoorthy, Alex A. Lemus, Adline Princy Solomon, Alex M. Valm, Prasanna Neelakantan

**Affiliations:** 1 Faculty of Dentistry, The University of Hong Kong, Pok Fu Lam, Hong Kong; akshmalakris@gmail.com; 2 Quorum Sensing Laboratory, Center of Research in Infectious Diseases, School of Chemical and Biotechnology, SASTRA Deemed to be University, Thanjavur 613401, India; adlineprinzy@sastra.ac.in; 3 Department of Biological Sciences, University at Albany, State University of New York, Albany, NY 12222, USA; alemus@albany.edu (A.A.L.); avalm@albany.edu (A.M.V.); 4 The RNA Institute, University at Albany, State University of New York, Albany, NY 12222, USA

**Keywords:** biofilm, *Candida albicans*, E-cadherin, *Enterococcus faecalis*, FISH, oral mucosa

## Abstract

*Candida albicans* as an opportunistic pathogen exploits the host immune system and causes a variety of life-threatening infections. The polymorphic nature of this fungus gives it tremendous advantage to breach mucosal barriers and cause oral and disseminated infections. Similar to *C. albicans*, *Enterococcus faecalis* is a major opportunistic pathogen, which is of critical concern in immunocompromised patients. There is increasing evidence that *E. faecalis* co-exists with *C. albicans* in the human body in disease samples. While the interactive profiles between these two organisms have been studied on abiotic substrates and mouse models, studies on their interactions on human oral mucosal surfaces are non-existent. Here, for the first time, we comprehensively characterized the interactive profiles between laboratory and clinical isolates of *C. albicans* (SC5314 and BF1) and *E. faecalis* (OG1RF and P52S) on an organotypic oral mucosal model. Our results demonstrated that the dual species biofilms resulted in profound surface erosion and significantly increased microbial invasion into mucosal compartments, compared to either species alone. Notably, several genes of *C. albicans* involved in tissue adhesion, hyphal formation, fungal invasion, and biofilm formation were significantly upregulated in the presence of *E. faecalis*. By contrast, E. faecalis genes involved in quorum sensing, biofilm formation, virulence, and mammalian cell invasion were downregulated. This study highlights the synergistic cross-kingdom interactions between *E. faecalis* and *C. albicans* in mucosal tissue invasion.

## 1. Introduction

*Candida albicans* resides as a commensal organism in the human microbiota and exists in homeostasis with the microbial flora and epithelial tissues in healthy individuals [1]. However, it takes advantage of a weakened immune system and generates disturbances in such equilibrium, leading to a variety of recalcitrant and life-threatening infections [2]. Oropharyngeal candidiasis and gastrointestinal candidiasis are common complications in patients who are on high dose cancer chemotherapy [3]. Here, *C. albicans* forms robust mucosal biofilms, invades them and causes tissue damage. The role of mucosal tissue biofilms in human disease is well-studied, and it is now known that persistent mucosal biofilms create conditions that may be conducive to recurrent infections. In particular, these biofilms allow for seeding of the microbes to distant locations in the body [4].

The oral cavity (or the upper gastrointestinal tract) is home to more than 700 different bacterial species. These bacteria form organized communities called biofilms that are attached on both the soft and hard tissues, as well as artificial prosthesis in the mouth. In the soft tissues, host immune responses as well as epithelial turnover limit this biofilm overgrowth. Furthermore, *C. albicans* colonization in mucosal sites is also impeded by the resident (commensal) bacterial community. However, studies have demonstrated that immunosuppressive conditions alter the dynamic equilibrium between microbiota, resulting in dysbiosis. Here, overgrowth of specific bacterial species that establish mutualistic interactions with candida species results in increased tissue damage and invasion. Such mutualism has been demonstrated extensively between *C. albicans* and commensal oral Streptococci in both in vitro and mouse models. For example, *S. oralis,* which alone is incapable of forming mucosal biofilms, forms hypervirulent mucosal biofilms when co-infected with *C. albicans* [5]. Therefore, it has been suggested that studies on mucosal biofilms independent of abiotic surface biofilms is critical to the understanding of disease pathogenesis and the development of novel anti-microbial drugs [6].

*Enterococcus faecalis*, a Gram-positive bacterium, is known to cause several infections including endocarditis, bacteremia, abdominal abscesses, burn wound sepsis, meningitis, urinary tract infections, and various nosocomial infections, particularly in immunocompromised patients. This organism holds a certain clinical relevance as it is resistant to multiple antibiotics. Although *E. faecalis* can form discrete microcolonies on the epithelial surface [7], it is usually considered to be a transient commensal which does not penetrate oral mucosal barriers [3]. Recent robust investigations on abiotic substrates showed that *E. faecalis* OG1RF was antagonistic to *Candida albicans* SC5314 on abiotic surfaces [8]. By contrast, there is mounting evidence that *E. faecalis* and *C. albicans* co-exist in several human disease samples including tongue mucosal infections [9], sputum, sepsis [10], and root canal infections [11]. In particular, the oral carriage of *E. faecalis* in healthy adults (< 10%), increases by about six-fold in immunocompromised patients such as those with cancer or diabetes, where oropharyngeal candidiasis is very common [3,9,12]. It has also been hypothesized that *E. faecalis* species in the root canals likely originate from the oral cavity sources such as saliva [13]. More recently, using immunocompromised mouse models, it was shown that *C. albicans* infection influenced the bacterial composition of the oral mucosa, dominated by mouse indigenous *E. faecalis* isolates [3]. Furthermore, *E. faecalis* depletion in antibiotics treated mice attenuated *C. albicans* invasion [3], indicating that the former was required to invade mucosal tissues by the latter.

Therefore, there is certain evidence to demonstrate synergy between these two microbes to cause dysbiosis on oral mucosa of mice. However, the role of clinical isolates of *E. faecalis* in enhancing *C. albicans* mucosal infections has never been examined. Here, we hypothesized that *C. albicans* and *E. faecalis* act mutually and form robust mucosal biofilms, which cause tissue destruction. Using a variety of comprehensive investigations, we demonstrate that characterize the biofilm formation, microbial invasion, and tissue destruction in mono-species and dual-species infections on commercial reconstructed human oral epithelia.

## 2. Materials and Methods

### 2.1. Chemicals, Microbial Strains, Engineered Tissue, and Culture Conditions

The microbial strains used in this study were human clinical isolates from our archival collection of the Central Research Laboratory, Faculty of Dentistry, The University of Hong Kong and well-characterized, widely used reference strains (*E. faecalis* OG1RF and *C. albicans* SC5314). *E. faecalis* OG1RF was originally isolated from the oral cavity, while *C. albicans* SC5314 was originally isolated from human infections (http://www.candidagenome.org/Strains.shtml#SC5314), although the exact origin remains unclear. The clinical isolates *E. faecalis* P52S and *C. albicans* BF1 were isolated from the oral cavity and have been discussed in detail elsewhere [13,14,15]. *E. faecalis* strains were grown overnight in Brain Heart Infusion Broth (BHI) under static conditions at 37 °C, while *C. albicans* strains were grown overnight in Yeast Potato Dextrose Broth (YPD) aerobically at 25 °C. The YPD medium consist of 5 g/L of yeast extract, 10 g/L of peptone, and 20 g/L of dextrose.

Reconstructed Human Oral Epithelium (RHOE) along with its maintenance media commercially obtained from MatTek Corporation (Ashland, Massachusetts, USA) were used in all the experiments. The assays were performed by growing the strains to mid-logarithmic phase and the OD595 was adjusted to achieve ~1.5 × 10^7^ CFU/mL based on extensively optimized methods in our laboratory using colony counting assays on selective agar media. The same culture conditions were maintained for all the experiments. All experiments were performed in triplicates as three independent experiments. Both the clinical isolate combinations and reference strain combinations were used in all experiments except the lactate dehydrogenase assay and the gene expression studies (where only the reference strains were used).

### 2.2. Inoculation of Mucosal Tissues with C. albicans and E. faecalis

Overnight cultures of *E. faecalis* and *C. albicans* were washed with phosphate buffered saline (PBS) and resuspended in fresh media. Mucosal tissues were transferred to 6 well plates containing 0.9 mL of pre-warmed MatTek assay medium per well. The microbial inoculum was prepared by inoculating 10^7^ cells each of *C. albicans* and *E. faecalis* in PBS. 60 µL of this inoculum was inoculated on the upper surface of the tissues and incubated for 18 h. For certain assays, the tissues were incubated for 48 h. The media was then collected, and the tissues were fixed with 10% formalin. Uninfected tissues were used as control in all experiments.

### 2.3. Analysis of Mucosal Biofilms and Microbial Invasion

Formalin fixed tissues were dehydrated using a series of ethanol and xylene concentrations and then embedded in paraffin as described previously [16]. Tissue sections of 10µm thickness were obtained using a microtome and the sections were mounted on polysine slides (Rotary Microtome, Leica Rm2155, Wetzlar, Germany).

#### 2.3.1. Hematoxylin and Eosin Staining

The slides mounted with the tissue sections were deparaffinized and stained with Haematoxylin and Eosin. The stained sections were visualized under the light microscope to observe the tissue architecture, biofilm formation, and the invasion through mucosal layers [16].

#### 2.3.2. Fluorescent in situ Hybridisation (FISH) and Quantification of Tissue Invasion by Microbial Isolates

Sections were labelled with fluorescence in situ hybridization probes as follows. Sections were labelled simultaneously with Caal probe for *C. albicans*, conjugated to Alexa fluor 488 (5′-GCCAAGGCTTATACTCGCT-3′) [17] and EUB 338 probe for *E. faecalis* conjugated to Alexa fluor 594 (5′-GCTGCCTCCCGTAGGAGT-3′) [18]. Custom oligonucleotide probes were synthesized by Thermo Fisher Scientific (Waltham, MA, USA). FISH was carried out according to previously published protocols [19], except the hybridization time, which was extended to 8 h based on our pilot studies for optimization. Hybridization solution [900 mM NaCl, 20 mM Tris, pH 7.5, 0.01% SDS 20% (vol/vol) formamide] and both probes at a final concentration of 2 nM] were applied to the samples and incubated at 46 °C for 8 h in a hybridization chamber. Slides were washed in wash buffer 1 [900 mM NaCl, 20 mM Tris, pH 7.5, 0.01% SDS 20% (vol/vol) formamide] at 48 °C for 15 min, then with wash buffer 2 [900 mM NaCl, 20 mM Tris, pH 7.5, 0.01% SDS] at 48 °C for 15 min, then dehydrated in an ethanol series, mounted in ProLong Gold antifade mountant with the blue fluorescent DNA stain (4′,6-diamidino-2-phenylindole (DAPI, Thermo Fisher Scientific, Waltham, MA, USA) and covered with a #1.5 coverslip. After curing overnight in the dark, the slides were imaged using a Zeiss LSM 710 confocal microscope with 20x 0.8 NA objective. Spectral images were acquired and then unmixed using the Zeiss Zen software and reference spectra were extracted from the images as described previously [20].

Quantitative analysis of mucosal biofilms was performed as described previously [21]. For each condition, the number of microbial cells, *E. faecalis* or *C. albicans*, was quantified in each field of view using the Imaris software. Each microbial cell was segmented using a local threshold set in the software. The distance of each microbial cell to the well-defined basement membrane was measured. The apical tissue surface was defined as the mean distance between the apical epithelial cells that were closest to the basement membrane and open to the external surface and the most apical epithelial cells. Tissue autofuorescence served as a well-defined marker for these epithelial cells. Any microbe that was located below this mean distance from basement membrane was considered invasive and any cell at or above this threshold distance was considered non-invasive. Then, the microbial cells below the tissue surface (the “invasive” microbes) were counted and then compared to the fraction of microbial cells located on the epithelial surface as described previously.

### 2.4. Evaluation of Tissue Integrity Using e-cadherin

Immunofluorescent staining of E-cadherin was performed to evaluate the integrity of the mucosal layer challenged by the biofilms [22]. Deparaffinized sections were washed with PBS and antigen retrieval was achieved by treating the specimens with Saponin for 20 min at 95 °C, cooled slowly and washed with PBS for 10 min. The sections were blocked using 10% normal goat serum for 30 min at room temperature, then incubated with mouse monoclonal primary antibody against E-cadherin at 37 °C for 1.5 h (dilution ratio of 1:50). Subsequently, the unbound primary antibodies were removed by washing with PBS and the slides were incubated with the secondary antibody (Alexa Fluor 488 goat anti-mouse IgG) for 1 h at room temperature under dark conditions.

The samples were then mounted with a Fluoro-Gel II mounting medium containing DAPI. Slides were imaged using a Zeiss LSM 710 confocal microscope with 63x 1.4 numerical aperture objective. Spectral images were acquired then unmixed using Zeiss Zen software and reference spectra extracted from the images as described above. Tissue autofluorescence spectral signatures were acquired from images of unstained tissue sections and subtracted from the images.

### 2.5. Quantification of Tissue Destruction Using the Lactate Dehydrogenase (LDH) Assay

The Lactate dehydrogenase (LDH) released into the basal culture media was monitored as an indicator of tissue/cell damage [1,16]. Here, biofilms of the reference strains were developed on mucosal tissues using standardized cell numbers for the mono-and dual species infections so that the total cell number was the same in all the groups. After 48 h, the supernatant was collected from the culture medium of the infected tissues and LDH activity was measured using a LDH cytotoxicity assay kit (Cayman Chemical Company, Ann Arbor, MI, USA) by measuring the OD490 and OD680 using a microplate reader (Spectramax M2 and M2e Multi-Mode Microplate Reader, Molecular Devices, San Jose, CA, USA). Media from the uninfected tissues served as controls.

### 2.6. Gene Expression Studies

Real-Time PCR (RT-PCR)-based transcriptomic studies were performed to analyze the gene-expression changes in mucosal biofilms of the reference strains. The tissue sections were homogenized using PBS to remove the loosely adhered cells. Total RNA was extracted using the SV total RNA isolation system (Promega, Madison, WI, USA). The integrity and purity of total RNA was assessed by NanoDrop (Nanodrop 2000c, Thermo Fisher Scientific). cDNA was synthesized using the High-Capacity cDNA Reverse Transcription kit (Applied Biosystems, Foster City, CA, USA). Expression changes of the genes involved in biofilm formation, hyphal formation, virulence, and metabolism were analyzed using RT-PCR (Real-Time Pcr System, Stepone™ & Steponeplus™, Applied Biosystems). Details of the primers and the standard reporting of the RT-PCR reactions have been tabulated in the Supplementary materials. 18srRNA and 23srRNA were used as the reference genes for *C. albicans* and *E. faecalis* respectively. Fold changes in gene expression were calculated using the 2^-ΔΔCT^ method.

### 2.7. Statistical Analysis

All the assays were carried out in triplicates for three independent trials and the results were expressed as mean ± SD. Statistical analysis of the data was performed by one-way ANOVA (Graph Pad Prism version 6.05). Statistical analysis of the microbial invasion data from the FISH analysis was carried out using two-tailed Student’s t-tests. p≤0.05 was considered statistically significant.

## 3. Results and Discussion

### 3.1. Fungal Biofilms and Dual Species Biofilms cause Mucosal Tissue Erosion

**Figure 1:**
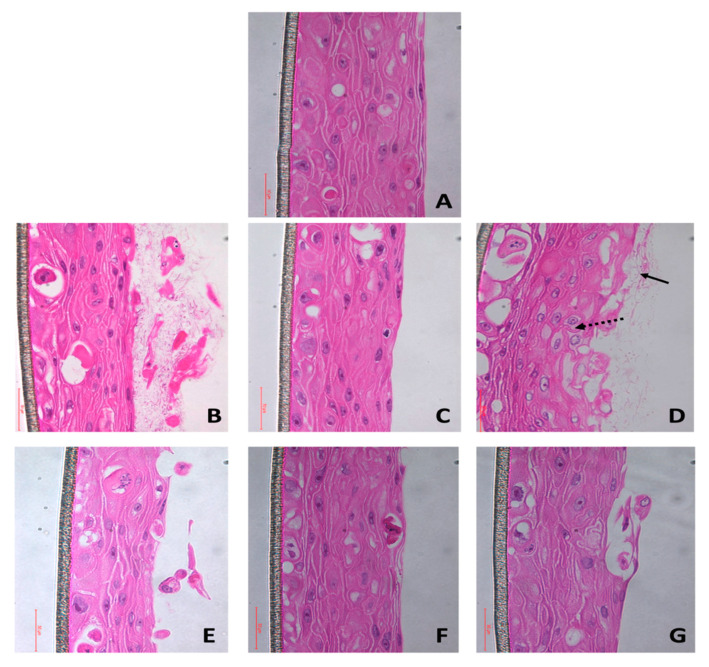
Cross-sectional images (x50) of the (**A**) uninfected control and (**B–G**) infected tissues stained with hematoxylin and eosin. Images B, C, and D represent tissues infected with *Candida albicans* SC5314, *Enterococcus faecalis* OG1RF, and *Candida albicans* SC5314 + *Enterococcus faecalis* OG1RF, respectively, while images E, F, and G represent tissues infected with *Candida albicans* BF1, *Enterococcus faecalis* P52S, and *Candida albicans* BF1 + *Enterococcus faecalis* P52S. The black arrow indicates the hyphae and the dotted arrow represents the loss in tissue integrity. Note that the tissues infected with the reference strain of C. albicans (SC5314) show dense and numerous hyphae, compared to the clinical strain (BF1), which does not demonstrate hyphal formation. Surface erosion of the mucosal tissues in all the tissues infected with C. albicans (B, D, E, and G) is apparent, compared to the control (A).

It has been shown previously that *E. faecalis* attenuates *C. albicans* hyphae formation on abiotic substrates [8,23]. We asked if the same interactive profiles would manifest on biotic substrates such as mucosal tissues. Invasion of *C. albicans* into the mucosal layer is promoted through several hyphal associated factors such as adhesion molecules (hyphal wall protein 1) and hydrolytic enzymes (phospholipases) [1]. *C. albicans* also interacts with the epithelia-associated proteins such as E-cadherin, which induces endocytosis and provides a mechanism for epithelial cell penetration [24]. Histological imaging of the infected tissues demonstrated that the surface erosion of tissues in the dual species biofilms of the reference strains (*E. faecalis* OG1RF and *C. albicans* SC5314) was similar to that observed with *C. albicans* alone, but greater than with *E. faecalis* alone (Figure 1). The surface biofilms showed large numbers of densely arranged hyphae in the mono-species *C. albicans* and dual species challenged mucosa for the reference strains. By contrast, *E. faecalis* alone did not expand into large, robust mucosal biofilms, although it did form microcolonies, indicative of attachment but not subsequent growth [25], which corroborated with our hematoxylin and eosin staining results.

We then asked if these results were true for human clinical isolates. Interestingly, *Candida albicans* BF-1 showed neither dense biofilms nor hyphae in the mono-species as well as dual species models (Figure 1). Similarly, *E. faecalis* P52S, which only colonized in low cell density, was neither invasive nor destructive as the OG1RF strain. These results are in contrast with the findings of Graham et al. [13], who demonstrated that *E. faecalis* inhibited hyphal formation by *C. albicans* on abiotic substrates. Furthermore, the authors purified the peptide responsible for such effects and tested their potent antifungal effects in animal model. These contrasting results observed between biotic and abiotic surfaces are noteworthy and scientifically important as they demonstrate the substrate-dependency and strain-dependency of microbial interactions.

### 3.2. Dual Species Biofilms Demonstrate Increased Microbial Invasion into Mucosal Compartments than Mono-Species Biofilms

**Figure 2:**
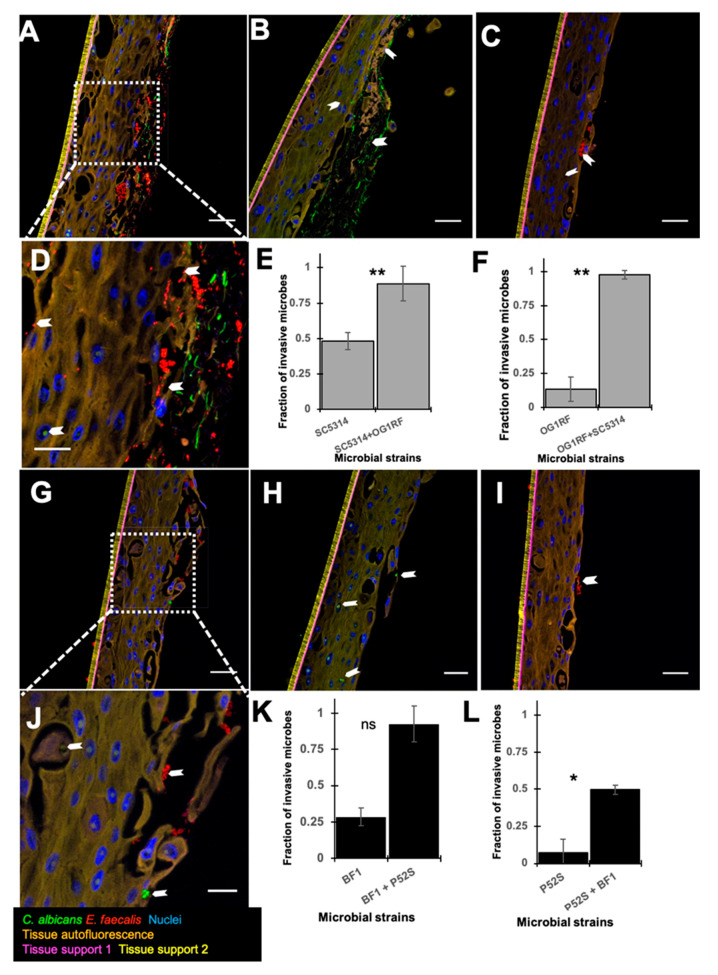
Analysis of microbial invasion in an in vitro organotypic oral mucosal model. All images are spectral fluorescence images. *C. albicans* (green) was visualized after staining with Caal probe conjugated to Alexa fluor 488 and *E. faecalis* (red) was visualized after staining with a EUB 338 probe conjugated to Alexa fluor 594. In the multispectral fluorescent in situ hybridisation (FISH) images, tissue autofluorescence is pseudocolored orange and tissue substrate materials are pseudocolored magenta and yellow. White arrows indicate bacterial/fungal cells that invaded into the mucosal tissue layers. (**A**) Image of co-infection model with the reference strains *C. albicans* SC5314 and *E. faecalis* OG1RF; (**B**) Image of monoculture infection with *C. albicans* SC5314; (**C**) Monoculture infection with *E. faecalis* OG1RF; (**D**) Zoomed image of region of interest within dotted box in panel A showing clear evidence of tissue invasion by both *E. faecalis* and *C. albicans*; (**E**) Quantification of tissue invasion by *C. albicans* SC5314 in monoculture vs. co-culture models; (**F**) Quantification of tissue invasion by *E. faecalis* OG1RF in monoculture and co-culture models; (**G**) Image of co-infection model with clinical strains *C. albicans* BF1 and *E. faecalis* P52S; (**H**) Image of monoculture infection with *C. albicans* BF1; (**I**) Monoculture infection with *E. faecalis* P52S; (**J**) Zoomed image of region of interest within dotted box in panel G showing tissue invasion by both *E. faecalis* and *C. albicans*, though to a lesser extent than observed with the reference strains as visualized in panel D; (**K**) Quantification of tissue invasion by *C. albicans* BF1 in monoculture and co-culture models; (**L**) Quantification of tissue invasion by *E. faecalis* P52S in monoculture vs. co-culture models. Scale bars in A–C and G–I = 50 µm. Scale bars in D & J = 20 µm. Bars in E, F and K, L represent mean values from 3 technical replicates. Error bars represent standard deviation. ** = *p* ≤ 0.01, * = *p* ≤ 0.05, ns = *p* > 0.05.

Microbial invasion into mucosal tissues was qualitatively and quantitatively investigated using the FISH assay and computational analysis of the images (Figure 2). Here, the distance from the distinctive and reliable epithelial basement marker to each microbial cell was calculated. Then, the distinctive autofluorescence signal from the epithelial cell layer was used to identify the apical border of the tissue. The number of microbial cells located apical to the tissue boundary or sub-apical was then quantified. These analyses demonstrated that in dual species biofilms, both *C. albicans* and *E. faecalis* invaded the mucosal compartments in significantly larger fractions than the corresponding mono-species infections (Figure 2), with the exception of the *C. albicans* clinical isolate which invaded in moderately greater but statistically insignificant numbers in dual species biofilms, compared to the mono-species infections. These notable findings confirm mutualistic interactions between the two organisms in tissue invasion. Although the majority of *E. faecalis* cells appear to be located extracellularly within disrupted epithelial tissues, the presence of intracellular bacterial cells cannot be ruled out with the level of resolution achieved here. Future studies with super resolution imaging combined with FISH are needed to rule out the presence of intracellular invasion of *E. faecalis* in this model.

**Figure 3:**
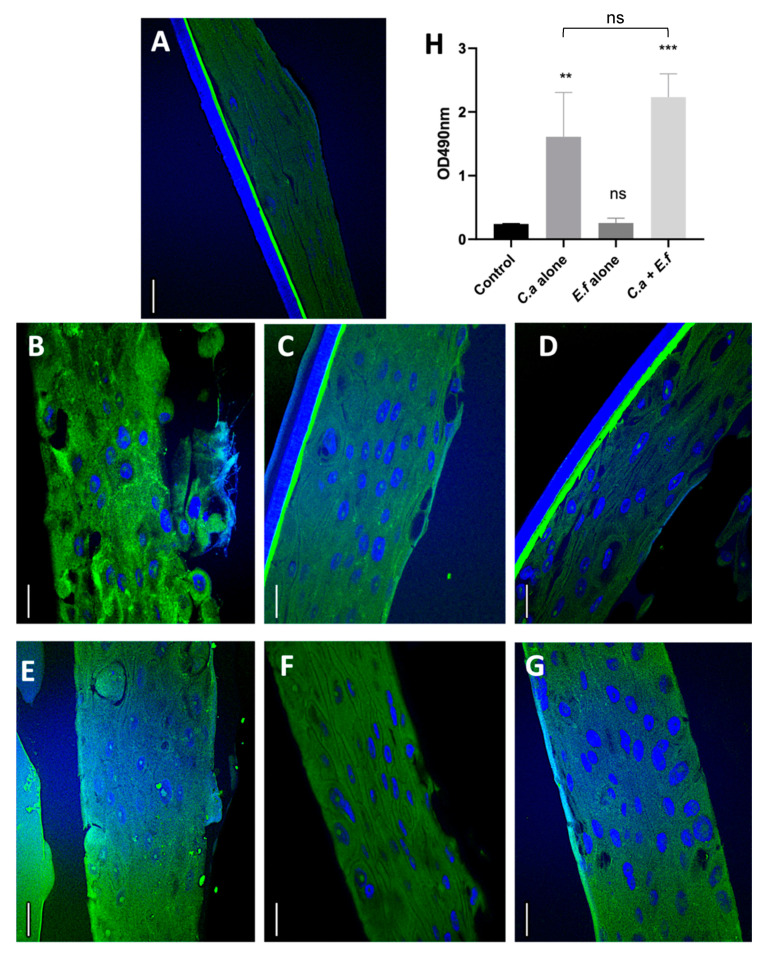
Immunofluorescence staining of E-cadherin in (**A**) uninfected control and (**B–G**) infected tissues in an organotypic oral mucosal model. Thee immunofluorescence staining was performed using monoclonal antibody conjugated with Alexa Fluor 488 (green) and are counterstained with the nucleic acid stain DAPI (blue). Images B, C, and D represent tissues infected with *Candida albicans* SC5314, *Enterococcus faecalis* OG1RF, and *Candida albicans* SC5314 + *Enterococcus faecalis* OG1RF, respectively, while images E, F, and G represent tissues infected with *Candida albicans* BF1, *Enterococcus faecalis* P52S, and *Candida albicans* BF1 + *Enterococcus faecalis* P52S. Note that the tissues infected with the reference strain of *C. albicans* SC5314 (panel B) and *C. albicans* SC5314 + *E. faecalis* OG1RF (panel D) loss of tissue integrity on the mucosal surface and sub-surface. Loss of integrity is also evident in tissues infected with the clinical strain of *C. albicans*, though to a lesser extent than observed with the reference strain as visualized in panel B. Scale bars in A–C and G–I = 25 µm. Graph in panel **H** shows results of the Lactate dehydrogenase (LDH) assay indicating a moderate but insignificant increase in LDH released in the dual-species infected tissues compared to the *C. albicans* infected tissues. ** denotes *p* ≤ 0.01 and *** denotes *p* ≤ 0.001, and ns denotes *p* > 0.05. *C.a*—*C. albicans*; *E.f*—*E. faecalis*.

*C. albicans* adheres to and invades epithelial tissues by inducing endocytosis. To achieve this, the fungal hyphae express specific invasin-like molecules that bind to host cell receptors such as E-cadherin [26]. E-cadherin, a calcium dependent homophilic molecule, is important for cell–cell adhesion in epithelial tissues [22,23]. *C. albicans* invades into intercellular compartments in epithelial tissue by proteolytically degrading E-cadherin [27,28]. To identify the potential mechanisms by which dual species biofilms caused increased tissue destruction, we asked if these biofilms degraded E-cadherin significantly more than mono-species biofilms. Qualitative investigations indicated increased degradation of E-cadherin in the *C. albicans* SC5314 mono-species as well as dual-species infections when compared with the uninfected control tissues and the *E. faecalis* infected tissues (Figure 3). This may be attributed to the increased hyphal invasion into mucosal compartments in the dual species biofilm, as shown by the FISH analysis.

Based on these findings we questioned if the tissue destruction can be demonstrated quantitatively. Therefore, the tissue destruction was quantified by assessing the supernatant using the LDH assay. Increased LDH amounts indicate more tissue destruction. Pilot studies using colony forming unit assays (data not shown) showed that *E. faecalis* did not affect the growth of *C. albicans*. Furthermore, the same number of cells were used for the mono-and dual-species infections. Therefore, the results of the LDH assay can be correlated with the tissue destruction. Dual species biofilms were characterized by significantly greater LDH quantity than *E. faecalis*, but not *C. albicans* mono-species infections. The clinical isolate of *C. albicans* and dual species biofilms of the clinical isolates demonstrated E-cadherin degradation, although this was markedly less than the observations with the reference strains. Further studies are required to quantify the tissue destruction with several clinical isolates using approaches such as Western Blot. Taken together, while *E. faecalis* may not enhance the mucosal tissue damage by *C. albicans*, it is evident that the two species are synergistic in invading mucosal tissues. This corroborates with the mounting evidence that *E. faecalis* and *C. albicans* promote a mutually beneficial association in human disease sites [29].

### 3.3. E. faecalis Upregulates the Expression of Selected Virulence Genes of C. albicans

**Figure 4:**
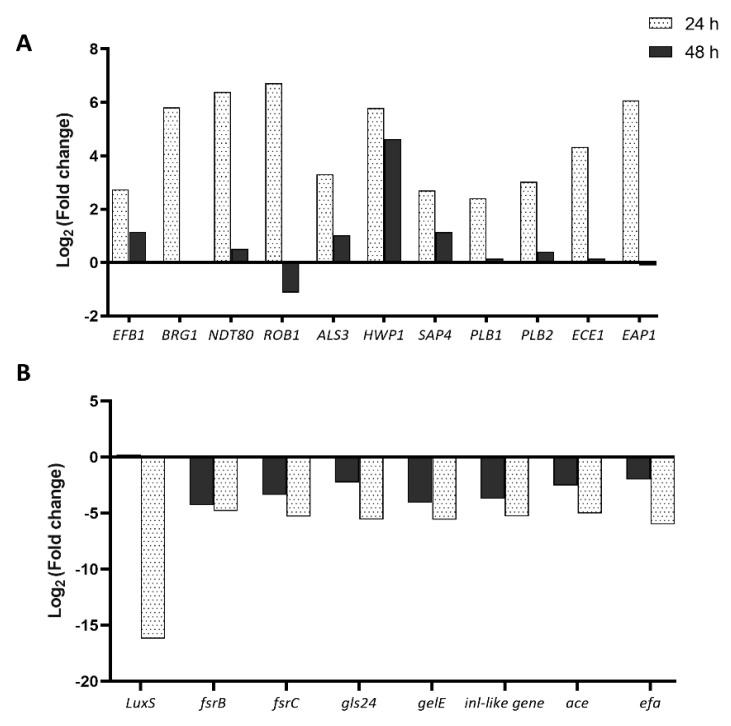
Real-time PCR (RT-PCR) analyses of different master regulatory genes present in the reference strain. (**A**) Logarithmic relative change of the gene expression levels in the dual species mucosal model, relative to the *C. albicans* mono-species infections at 24 and 48 h with 18srRNA as housekeeping gene; (**B**) Logarithmic relative change of the gene expression levels in dual species mucosal model relative to the *E. faecalis* mono-species infections at 24 and 48 h with 23srRNA as housekeeping genes.

Our experiments on mucosal tissue invasion demonstrated that *E. faecalis* was synergistic with *C. albicans*. As described previously, the number of hyphae in the dual-species biofilms of the reference strain appeared reduced compared to the mono-species infections, although deeper invasion of the fungi and larger number of invading cells were observed. Furthermore, tissue destruction in the dual-species biofilms was marginally and insignificantly greater than *C. albicans* mono-species infections indicating that *E. faecalis* did not attenuate *C. albicans*. To further explain the interactions, we tested the expression levels of some carefully selected genes that are highly relevant to mucosal tissue biofilm formation and invasion in dual species mucosal biofilms, relative to the mono-species biofilms. Specifically, as shown in the Tables S1 and S2, this included *C. albicans* genes regulating biofilm formation, adhesion, tissue invasion, hyphal formation, and production of enzymes that facilitate fungal tissue invasion. For *E. faecalis*, genes that regulate inter-species communication, biofilm formation, production of virulence factors, and tissue invasion were investigated. The results revealed that *E. faecalis* dramatically upregulated (6-fold) *ROB1, NDT80*, and *BRG1*, the master regulatory genes involved in adhesion and biofilm formation in *C. albicans* [28], indicating that the former enhanced the ability of the latter to adhere to epithelial tissues and facilitated its biofilm growth (Figure 4). Rather interestingly, gene expression of all the genes was downregulated at 48 h when compared to 24 h, which suggests that these genes are involved only in the early stage of infection or only during biofilm formation. It is also possible that these genes were not upregulated due to the complete tissue destruction at 48 h.

After the adherence phase, *C. albicans* invades epithelial tissue by inducing endocytosis, by expressing invasions such as *ALS3*, which is regulated by the gene *ALS3*. This gene was upregulated by ~3-fold in the dual species infections, suggesting that *E. faecalis* potentially enhanced the endocytosis of *C. albicans* [26]. Both the E-cadherin and the FISH assays demonstrated hyphal invasion into mucosal compartments in the dual species biofilm [22]. Correspondingly, RT-PCR analysis of the hyphal wall protein 1 (*HWP1*) showed an upregulation in dual-species biofilm model relative to *C. albicans* mono-species biofilms (Figure 4). However, the number of hyphae in the dual-species biofilms appeared less than in the mono-species fungal biofilms. Despite that, the tissue invasion (as shown by the FISH assay) and tissue destruction (as shown by the LDH assay) in the dual-species infection was comparable to *C. albicans* infections. Upregulation of the genes *PLB1*, *PLB2*, and *SAP4* demonstrated that further studies may be required to confirm that it was mediated by the production of fungal aspartyl proteases and phospholipases [26,30]. Such effects have been reported for *S. oralis*–*C. albicans* biofilms [27]. These results corroborate with the findings from our E-cadherin assay as described previously. Taken together, our results indicate that *E. faecalis* supports the tissue degradation and invasion *C. albicans* by modulating its virulence genes.

Interestingly, all the tested genes of *E. faecalis* in the dual species biofilms were downregulated compared to the mono-species biofilm. The two-component regulatory system consisting of the *fsr* locus in *E. faecalis* is critical for establishing its virulence. *gelE, sprE, ef1097,* and *ef1097b* are the four major genes that are directly dependent on the Fsr system, governing the production of gelatinase and serine protease [31,32]. Several post translational modifications of the genes *gelE* and *sprE* are required for activating EntV [31,32]. EntV, a bacteriocin produced by *E. faecalis* suppresses hyphal morphogenesis of *C. albicans* in vitro [8,23]. While this is true for abiotic substrates (e.g., dentures or catheters), our results demonstrate mutualistic interactions on mucosal tissue substrates. Expression of *gelE, fsrB*, and *fsrC* which are involved in the production of EntV were downregulated (5-fold). The *inl-like gene* of *E. faecalis* is responsible for the invasion of mammalian cells via E-cadherin. Our results showed a significant downregulation of this gene, explaining why *E. faecalis* alone was unable to destruct tissues or invade mucosal compartments. The marked upregulation of many *C. albicans* genes responsible for tissue destruction and invasion in the presence of *E. faecalis*, indicates that these organisms act mutually on the mucosal surface and are not antagonistic to each other.

Although we have provided the first evidence on the role of *E. faecalis* in *C. albicans* tissue invasion and surface erosion, only a limited number of carefully selected strains were tested in this study. Future studies will be performed with more clinical isolates. Furthermore, we tested a series of pertinent virulence genes for the qRT-PCR. Global transcriptomic responses in dual species mucosal biofilms and the ensuing mucosal inflammatory responses will be investigated in future studies.

## 4. Conclusions

This study shines significant new light on the interactive profiles between reference strains and clinical isolates *E. faecalis* and *C. albicans* on mucosal substrates. Dual species biofilms of the reference and clinical strains were mutualistic in increasing the tissue invasion of both the fungal and bacterial cells. Tissue destruction and surface erosion in the dual species biofilms were similar to those resulting from *C. albicans* infection alone. *E. faecalis* upregulated several *C. albicans* genes responsible for tissue adhesion, biofilm and hyphae formation, and invasion, while genes governing biofilm formation, virulence, and tissue invasion in *E. faecalis* were downregulated. These novel findings strongly suggest that multi-species biofilm communities should be investigated on a variety of substrates and environments.

## Supplementary Materials

The following are available online at https://www.mdpi.com/2076-2607/8/11/1771/s1, Table S1: Primer sequences of *Candida albicans* for RT-qPCR analysis, Table S2: Primer sequences of *Enterococcus faecalis* for RT-qPCR analysis, S3 Materials: Reporting of RT-qPCR conditions.
