# Supplementary material for: Interactions between Candida albicans and Enterococcus faecalis in an Organotypic Oral Epithelial Model
Source: Microorganisms. 2020 Nov 11;8(11):1771. doi: 10.3390/microorganisms8111771 (PMC7696566; doi:10.3390/microorganisms8111771)
Supplement: Supplementary file 1 [file microorganisms-08-01771-s001.pdf]

Supplementary Materials

Table S1. Primer sequences of *Candida albicans* for qRT-PCR analysis.

Genes	Primer Sequences	Reference	Key Roles
EFB1	F (5′AAGAAGGCTGCTAAAGGTCCA A3′)<br>R-(5′ATCCCATGGTTTGACATCCAA3′)	[1]	Master regulatory genes involved in adhesion and biofilm formation
BRG1	F(5′ACGATCAACCATTAGTGGAGG3′)<br>R(5′GAAGAAGTAGGTGTAGATGATCCAC3′)	[2]	
NDT80	F(5′CTCAACAAGGCCCAACACCTC3′)<br>R(5′TTGACGTGGTTGTCTTGCTGG3′)	[2]	
ROB1	F(5′CTGGATTCATCTCTTGGTTCACC3′)<br>R(5′TGTTGTTGGTTAAGGTGGACGTG3′)	[2]	
ALS3	F(5′CTGGACCACCAGGAAACACT3′)<br>R(5′ACCTGGAGGAGCAGTGAAAG3′)	[1]	Expression of invasion ALS3 causes epithelial tissue invasion by inducing endocytosis
HWP1	F(5′CGGAATCTAGTGCTGTCGTCTCT3′)<br>R(5′CGACACTTGAGTAATTGGCAGATG3′)	[1]	Hyphal wall protein helps in the attachment of hyphae to the host epithelial cells
SAP4	F(5′CGCTGGTGTCCTCTTAGATTCTG3′)<br>R(5′AGGCATAGATAATGCTACGAGCAA3′)	[1]	Production of fungal aspartyl proteases and phospholipases; host tissue invasion.
PLB1	F(5′GGTGGAGAAGATGGCCAAAA3′)<br>R(5′AGCACTTACGTTACGATGCAACA3′)	[1]	
PLB2	F(5′TGAACCTTTGGGCGACAACT3′)<br>R(5′GCCGCGCTCGTTGTTAA3′)	[1]	
ECE1	F (5′GTCGTCAGATTGCCAGAAATT G3′)<br>R(5′CTTGGCATTTTCGATGGATTGT3′)	[1]	Cell elongation and biofilm formation
EAP1	F(5′TGTGATGGCGGTTCTGTTC3′)<br>R(5′GGTAGTGACGGTGATGATAGTGACA3′)	[1]	Surface adhesion and hyphae development

Table S2. Primer sequences of *Enterococcus faecalis* for qRT-PCR analysis.

Genes	Primer Sequences	Reference	Key Roles
luxS	F(5′AACGTCCCAATTCTCTGCGT3′)<br>R(5′CGCTGACAGGTTTTCCCTCT3′)	[3]	Quorum sensing system responsible for interspecies communication
fsrB	F(5′TGCTCAAAAAGCAAAGCCTTATAA3′)<br>R(5′GATGACGAGACCGTAGAGTATTACTGAA3′)	[4]	Quorum Sensing system; two-component regulatory system consisting of the fsr locus, critical for establishing virulence
fsrC	F(5′GCTTATTTGGAAGAACAACGTATCAA3′)<br>R(5′CGAAACATCGCTAGCTCTTCGT3′)	[4]	
gls24	F(5′TAACAGTCGATGGCGGCTTT3′)<br>R(5′CAGCGACTTGTTTTTACCAACTTC3′)	[4]	Virulence and general stress response
gelE	F(5′CGGAACATACTGCCGGTTTAGA3′)<br>R(5′TGGATTAGATGCACCCGAAAT3′)	[4]	Encodes gelatinase and contributes to virulence, the degradation of host tissues, and biofilm formation. Required for activating EntV, bacteriocin produced by *E. faecalis*
inl-like gene	F(5′GTGACAGTATTAGAGATCCGAGATTTG3′)<br>R(5′ATACGCAGGTGCTGTCTTAGATAA3′)	[4]	Responsible for the invasion of mammalian cells via E-cadherin.
ace	F(5′CGGCGACTCAACGTTTGAC3′)<br>R(5′TCCAGCCAAATCGCCTACTT3′)	[4]	Encodes protein that has characteristics of a bacterial adhesin
efa	F(5′TGGGACAGACCCTCACGAATA3′)<br>F(5′CGCCTGTTTCTAAGTTCAAGCC3′)	[4]	Helps in virulence by Conjugation and oxidative stress defense

References

1. Samaranayake, Y.H.; Cheung, B.P.; Yau, J.Y.; Yeung, S.K.; Samaranayake, L.P.; Human serum promotes Candida albicans biofilm growth and virulence gene expression on silicone biomaterial. *PLoS ONE*. **2013**, *8*, e62902. doi: 10.1371/journal.pone.0062902
2. Hnisz, D.; Bardet, A.F.; Nobile, C.J. et al. A histone deacetylase adjusts transcription kinetics at coding sequences during Candida albicans morphogenesis. *PLoS Genet.* **2012**, *8*, e1003118, doi: 10.1371/journal.pgen.1003118
3. Yang, Y.; Li, W.; Hou, B.; Zhang, C. Quorum sensing LuxS/autoinducer-2 inhibits Enterococcus faecalis biofilm formation ability. *J Appl Oral Sci.* **2018**, *26*, e20170566. doi:10.1590/1678-7757-2017-0566
4. Brett D.S.; Michael, S.G. Differential Expression of Virulence-Related Genes in *Enterococcus faecalis* in Response to Biological Cues in Serum and Urine. *Infection and Immunity*. **2002**, *70*, 4344–4352. doi:10.1128/IAI.70.8.4344-4352.2002.

S3 Materials. Reporting of qRT-PCR conditions

cDNA conversion

Reaction setup/reaction:

Reagents	Volume
10X RT buffer	2 μL
25X dNTP Mix	0.8 μL
10X RT Random primers	2 μL
Reverse transcriptase	1 μL
Nuclease-free H_2O	4.2 μL
Total	10 μL

Thermal Cycling conditions

Settings	Step 1	Step 2	Step 3	Step 4
Temperature	25 ˚C	37 ˚C	85 ˚C	4 ˚C
Time (mins)	10	120	5	∞

qRT PCR conditions

Reaction setup/reaction

Reagents	Volume
SYBR Green	10 μL
Forward primer	1 μL
Reverse primer	1 μL
cDNA	1 μL
H_20	7 μL
Total	20 μL

Temperature conditions

Number of Cycles: 50

Stage	Temperature	Duration (min)
Holding Stage	95 ˚C	00:20
Cycling Stage	95 ˚C	00:03
	58 ˚C	00.30
Melt Curve stage	95 ˚C	00:15
	60 ˚C	1:00
	60 ˚C	00:15
